# Opposing global change drivers counterbalance trends in breeding North American monarch butterflies

**DOI:** 10.1111/gcb.16282

**Published:** 2022-06-10

**Authors:** Michael S. Crossley, Timothy D. Meehan, Matthew D. Moran, Jeffrey Glassberg, William E. Snyder, Andrew K. Davis

**Affiliations:** ^1^ Department of Entomology and Wildlife Ecology University of Delaware Newark Delaware USA; ^2^ National Audubon Society Boulder Colorado USA; ^3^ Department of Biology and Health Sciences Hendrix College Conway Arkansas USA; ^4^ North American Butterfly Association Morristown New Jersey USA; ^5^ Rice University Houston Texas USA; ^6^ Department of Entomology University of Georgia Athens Georgia USA; ^7^ Odum School of Ecology University of Georgia Athens Georgia USA

**Keywords:** conservation efforts, decline, glyphosate, monarch butterfly, population trends

## Abstract

Many insects are in clear decline, with monarch butterflies (*Danaus plexippus*) drawing particular attention as a flagship species. It is well documented that, among migratory populations, numbers of overwintering monarchs have been falling across several decades, but trends among breeding monarchs are less clear. Here, we compile >135,000 monarch observations between 1993 and 2018 from the North American Butterfly Association's annual butterfly count to examine spatiotemporal patterns and potential drivers of adult monarch relative abundance trends across the entire breeding range in eastern and western North America. While the data revealed declines at some sites, particularly the US Northeast and parts of the Midwest, numbers in other areas, notably the US Southeast and Northwest, were unchanged or increasing, yielding a slightly positive overall trend across the species range. Negative impacts of agricultural glyphosate use appeared to be counterbalanced by positive effects of annual temperature, particularly in the US Midwest. Overall, our results suggest that population growth in summer is compensating for losses during the winter and that changing environmental variables have offsetting effects on mortality and/or reproduction. We suggest that density‐dependent reproductive compensation when lower numbers arrive each spring is currently able to maintain relatively stable breeding monarch numbers. However, we caution against complacency since accelerating climate change may bring growing threats. In addition, increases of summer monarchs in some regions, especially in California and in the south, may reflect replacement of migratory with resident populations. Nonetheless, it is perhaps reassuring that ubiquitous downward trends in summer monarch abundance are not evident.

## Graphical abstract

A compilation of > 135,000 monarch observations between 1993‐2018 from the North American Butterfly Association’s annual butterfly count revealed declines at some sites, particularly the US Northeast and parts of the Midwest, while numbers in other areas, notably the US Southeast and Northwest, were unchanged or increasing, yielding a slightly positive overall trend across the species range. Negative impacts of agricultural glyphosate use appeared to be counterbalanced by positive effects of annual temperature, particularly in the US Midwest. We suggest that density‐dependent reproductive compensation when lower numbers arrive each spring is currently able to maintain relatively stable breeding monarch numbers.

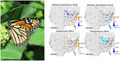

## 1 INTRODUCTION

Despite considerable variability through time, between sites, and among taxa, it is increasingly clear that some of the world's insects are in steep decline. This is perhaps best documented among bees and other pollinators, whose loss would have devastating consequences for global ecosystems and the human food supply (Wagner, 2020). Beyond pollination, insects are key providers of a full suite of provisioning, regulating, cultural, and supporting ecosystem services. Human degradation of the environment, at a range of scales, is often implicated in falling insect numbers (Fox, 2013; Habel, Samways, et al., 2019; Leather, 2018; Sánchez‐Bayo & Wyckhuys, 2019). A key local driver has been heavy herbicide and insecticide applications associated with agricultural intensification (Habel, Ulrich, et al., 2019). Urbanization and associated automobile collisions (Baxter‐Gilbert et al., 2015; Kantola et al., 2019) and light pollution bring additional challenges (Owens et al., 2020). At global scales, climate change can heighten physiological stress to insects while triggering spatiotemporal misalignment with, or reduced quality of, host plants or other resources (Bale et al., 2002; Jamieson et al., 2012), although even climate change can create variable regions of insect decreases and increases (Crossley et al., 2021; Koltz et al., 2018) such as when increased temperature enables faster population growth. Particularly damaging are cases where local and global drivers both are moving in harmful directions, for example when long‐distance migrants must move through increasingly hot and dry regions that also are seeing more intense land use (Saunders et al., 2019).

Monarch butterflies (*Danaus plexippus*) in North America have become the public face of insect declines (Gustafsson et al., 2015), largely because of the well‐publicized diminishing of winter colonies in Mexico and California (Boyle et al., 2019; Pelton et al., 2019). Monarchs are iconic insects due to their large size, attractive and distinctive coloration, wide range, host association with horticulturally popular milkweeds (*Asclepias* spp.), and fascinating long‐distance seasonal migrations. This has led to the prominent use of monarchs as ambassadors to engage the general public in insect conservation, for example, by facilitating the widespread planting of milkweed in home gardens (Thogmartin, López‐hoffman, et al., 2017). However, some of these same traits that make monarchs so charismatic to humans also subject the butterflies to particular risk. Best documented is habitat loss and changing climate at concentrated overwintering sites, which has apparently led to an ongoing, multi‐decadal decline of those colonies (Brower et al., 2012; Pelton et al., 2019; Thogmartin, Wiederholt, et al., 2017; Zylstra et al., 2021). A second widely‐touted threat is removal of milkweed from agricultural fields within the monarch's core breeding range in the American Midwest, following widespread adoption of glyphosate‐tolerant corn and soybean (Stenoien et al., 2018). Thirdly, since migration in the human‐dominated world is risky (Wilcove & Wikelski, 2008), their particularly long‐distance movements could expose monarchs to multiple threats along the 2 month journey (e.g., deaths from traffic collisions, Kantola et al., 2019; McKenna et al., 2001). Additionally, agricultural and residential pesticides (Olaya‐Arenas & Kaplan, 2019) and sensitivity to temperature and precipitation extremes as the climate changes (Lemoine, 2015; Saunders et al., 2018) may be adversely affecting monarchs at various stages of their life cycle.

Altogether, these perceived threats have led to the recent decision by USFWS that federal protection is warranted in the United States (USFWS, 2020). However, evidence is ambiguous whether monarchs continue to be in consistent, recent decline across the annual cycle (i.e., outside of the winter stage), with studies variously reporting steady or falling monarch numbers at different places and seasonal milestones (Brower et al., 2018; Davis & Dyer, 2015; Espeset et al., 2016; Ethier, 2020; Inamine et al., 2016; Ries et al., 2015). Uncertainty about whether breeding populations are continuing to steeply decline, or show some resiliency to overwintering losses in at least some regions or at some stages, complicates efforts to target conservation programs to points in the life‐cycle where they will be most effective.

Here, we used the North American Butterfly Association's (NABA) summer citizen‐science counts to assess spatiotemporal patterns and drivers of relative abundance of breeding, adult monarchs, and across most of their summer range throughout the United States (east and west) and southern Canada. Prior work with these or similar citizen‐science datasets have focused on specific regions of the country, such as the western US (Forister et al., 2021), or the Midwest (Zylstra et al., 2021). For a species like the monarch, which has a continental breeding range, it is important to assess the population throughout this large area, so that local or regional hotspots of decline or increase do not bias the interpretation of the entire population's status. These NABA data are broad in scope, collectively recording 135,705 monarchs at 403 sites across North America, over time periods of 10–26 years from 1993 to 2018. We analyzed NABA data using methods developed for a similar citizen‐science program, the Audubon Christmas Bird Count (Meehan et al., 2019), yielding monarch relative‐abundance trends that accounted for spatial and temporal variation in sampling effort as well as spatial and temporal autocorrelation among neighboring counts. Our central goals were to (1) quantify trends in monarch relative abundance among NABA sites throughout the United States and southern Canada, and (2) characterize relationships between those trends and two dominant global change factors: agricultural intensification, specifically glyphosate use, and climate change, specifically temperature and precipitation change.

## 2 METHODS

### 2.1 Butterfly data

We used direct counts of monarch adults from the North American Butterfly Association's summer citizen‐science counts (https://www.naba.org/). Butterfly counts are made within a 15‐mile (~24 km) diameter circle, typically in July, and are open to participation from the public. For each count event, the abundances of butterfly species are tallied and the sum of associated party hours (a measure of sampling effort that aggregates the number of hours spent by each observer) is recorded. To minimize bias due to differences among sites in the day of year when butterfly counts were conducted, we limited our analysis to butterfly counts that occurred between June 1 and August 31. Prior to estimating trends in abundance, we removed sites that had <5 years of monarch detections and that spanned <10 years (Didham et al., 2020). Lastly, butterfly counts were assigned to 50 × 50 km (2500‐km^2^) cells on a uniform grid covering North America to enable spatial smoothing of estimated relative abundance trends and covariate effects. Grid cells contained an average of 1.21 ± 0.03 and a maximum of three circles. The curated dataset recorded a total of 135,705 monarchs from 403 sites occupying 334 grid cells, over time periods of 10–26 years from 1993 to 2018.

### 2.2 Modeling relative abundance trends

We modeled monarch counts, $y_{i,k,t}$, in grid cell i encompassing count circle k during year t as a random variable from a negative binomial distribution. Expected values for counts per grid cell, $\mu_{i,t}$, were assumed to be a function of spatially structured grid‐cell, count‐effort, and year effects, plus unstructured variation among count circles. The linear predictor for $\mu_{i,t}\;$ took the form

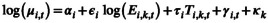

Parameters $\alpha_{i}$ were modeled as cell‐specific random intercepts with an intrinsic conditional autoregressive (iCAR) structure. Parameters $\epsilon_{i}$ were modeled as spatially structured (iCAR), cell‐specific, random slope coefficients for the local effects of effort $E_{i,k,t}$. Effort was represented by $E_{i,k,t}$, the number of party hours expended during a count, where a party hour was the count effort of one party of unspecified size for 1 h. Pairing log‐transformed expected counts with log‐transformed effort in the linear predictor yielded a power function for effort correction, a flexible mathematical form that accommodated a decreasing, linear, or increasing impact of effort on expected counts (Link & Sauer, 1999). Parameters $\tau_{i}$ were modeled as spatially structured, cell‐specific, random slope coefficients for a log‐linear year effect. Year, represented by T, was transformed before analysis such that max(T) = 0, and each preceding year took an increasingly negative integer value. Given the scaling of effort and year variables, exp($\alpha_{i}$) could be interpreted as a cell‐specific expected count given one party hour of effort during the final year in the time series. Parameters $\gamma_{i,t}$ were modeled as exchangeable random intercepts that accounted for variation in relative abundance per grid cell and year that was not accounted for by the log‐linear year effect. The final term in the model, $\kappa_{k}$, was an exchangeable random intercept that accounted for variation in relative abundance among circles, possibly due to differences in habitat conditions or observer experience.

This spatially varying coefficient (SVC) model was analyzed within a Bayesian framework using the R‐INLA package in R (Lindgren & Rue, 2015; R Core Team, 2021; Rue et al., 2017). For parameters $\alpha_{i}$, $\epsilon_{i}$, and $\tau_{i}$, with iCAR structure (Besag et al., 1991), precision matrices were scaled such that the geometric mean of marginal variances was equal to one, and priors for precision parameters were penalized complexity (PC) priors, with parameter values $U_{\mathrm{PC}}=1$ and $a_{\mathrm{PC}}=0.01$ (Simpson et al., 2017). Precision for the zero‐centered, exchangeable, random circle effect, $\kappa_{k}$ and grid cell by year effect, $\gamma_{i,t}$, were also assigned a PC prior with parameter values $U_{\mathrm{PC}}=1$ and $a_{\mathrm{PC}}=0.01$ (Simpson et al., 2017). The overdispersion term for the negative binomial count distribution, Φ, was assigned a PC prior with parameter value l=7. (Rue et al., 2017).

Following trend model analysis, posterior medians and symmetric 95% credible intervals were computed per cell for $\alpha_{i}$, $\epsilon_{i}$, and $\tau_{i}$ and per cell and year for $\gamma_{i,t}$, by sampling the respective posterior distributions 5000 times. Posterior summaries were then mapped to visualize spatial variation in abundance indices, effort effects, and relative abundance trends.

### 2.3 Explaining spatiotemporal variation in relative abundance

The North American monarch breeding range spans nearly the entire United States and southern Canada, which includes widely differing landscapes (see Figure S1), including the heavily agricultural region in the Midwest. This region is where 38% of monarchs in Mexico come from (Flockhart et al., 2017), and this is where there has been significant losses of milkweeds due to application of glyphosate in crop fields (Brower et al., 2012). These losses have been proposed as one of the major reasons for the declines in winter colonies in Mexico, because of the temporal synchrony of glyphosate application and colony size decreases (Pleasants & Oberhauser, 2013). As such, determining the impact of glyphosate use on monarch abundance was a priority for us here. In addition, summer climate variables are also known to influence relative abundance of monarchs (Zylstra et al., 2021), and our analyses also incorporated such data.

We used posterior samples along with a subset of the linear predictor to calculate an annual relative abundance index, $\omega_{i,t}$, per year and grid cell, as $\omega_{i,t}=\exp\left(\alpha_{i}+\tau_{i}+\gamma_{i,t}\right)$. We then modeled relative abundance indices and their associated uncertainty for grid cell i during year t as a random variable from a gamma distribution. Expected values for annual abundance indices per grid cell, $\Omega_{i,t}$, were assumed to be a function of spatially structured grid cell, agricultural glyphosate use, average temperature, and cumulative precipitation effects (using data summarized below). The linear predictor for $\Omega_{i,t}$ took the form

$$\log\left(\Omega_{i,t}\right)=\beta_{i}+\rho_{i}P_{i,t}+\zeta_{i}Z_{i,t}+\nu_{i}N_{i,t}$$

**FIGURE 1:**
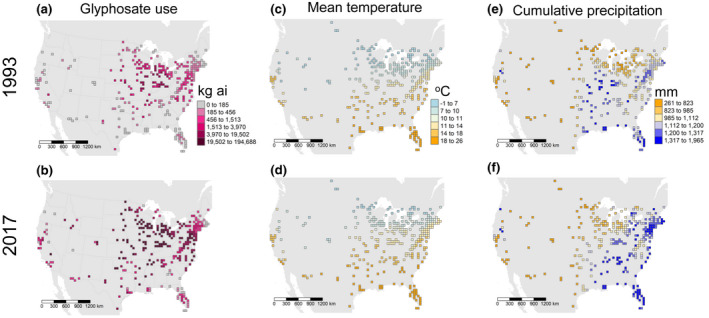
Maps of covariates considered in models of spatiotemporal patterns of monarch relative abundance in 1993 (earliest date when monarch data were available) and 2017 (latest year when glyphosate use data are available). (a, b) glyphosate use (kg active ingredient applied to corn and soybean). (c, d) Mean annual temperature. (e, f) Cumulative annual precipitation.

Parameters $\beta_{i}$ were modeled as cell‐specific random intercepts with iCAR structure. Parameters $\rho_{i}\;$ were modeled as spatially structured (iCAR), cell‐specific, random slope coefficients for the local effects of glyphosate use, $P_{i,t}$. Agricultural glyphosate use was calculated as the pounds of active ingredient applied in a county multiplied by the proportion of the county planted in corn or soybean, to account for the expectation that the majority of glyphosate use in a county that is negatively impacting monarch host plants is through applications to corn and soybean acreage (Zylstra et al., 2021). Estimates of pounds glyphosate applied were obtained from the United States Geological Survey—National Water‐Quality Assessment Project (USGS, 2022), and corn and soybean acreage were obtained from United States Department of Agriculture National Agricultural Statistics Service (USDA‐NASS, 2022) using the “rnassqs” R package (Potter, 2019). To obtain an estimate of agricultural glyphosate use for each grid cell × year, values of glyphosate use offset by the proportion corn or soybean in each county overlapping grid cell i in year t were multiplied by the proportion of overlap with grid cell i. Spatial operations were done in R using functions available from the “rgeos,” “raster,” and “rgdal” R packages (Bivand & Rundel, 2021; Bivand et al., 2021; Hijmans, 2022). Maps of glyphosate use (kg active ingredient per acre corn or soybean) in 1993 and 2017 are provided in Figure 1.

Parameters $\;\zeta_{i}$ were modeled as spatially structured (iCAR), cell‐specific, random slope coefficients for the local effects of mean annual temperature, $Z_{i,t}$. Parameters $\nu_{i}\;$ were modeled as spatially structured, cell‐specific, random slope coefficients for the local effects of cumulative precipitation, $N_{i,t}$. Mean temperature and precipitation data were obtained from CRU TS 4.03 (Harris et al., 2014), which provides monthly gridded estimates at 0.5° latitude/longitude resolution. Mean temperature for grid cell i in year t was calculated as the annual average of monthly mean temperature estimates in year t. Annual cumulative precipitation for grid cell i in year t was calculated as the sum of monthly precipitation estimates in year t. Maps of mean temperature and cumulative precipitation in 1993 and 2017 are provided in Figure 1.

To propagate uncertainty in relative abundance indices, $\omega_{i,t}$, during covariate analyses, the analysis was repeated 5000 times using randomly sampled values from the posteriors of *α*_*i*_, *τ*_*i*_, and *γ*_*i,t*_. Estimates for $\rho_{i}$, $\zeta_{i}$, and $\nu_{i}$ from each of the 5000 replicates were then used to generate posterior medians and symmetric 95% credible intervals per cell for $\rho_{i}$,$\;\zeta_{i}$, and $\nu_{i}$. Posterior summaries were then mapped to visualize spatial variation in covariate effects.

## 3 RESULTS

**FIGURE 2:**
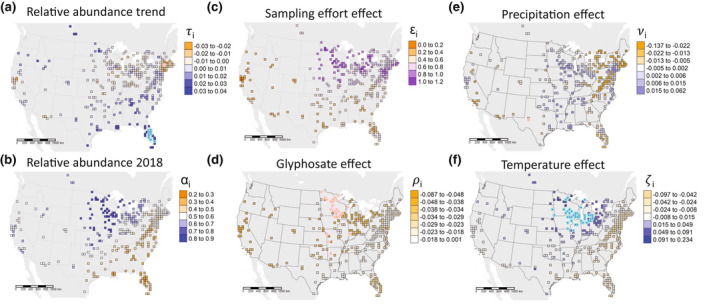
(a) Map of monarch relative abundance in 2018 (α_i_). (b) Map of sampling effort effect (ε_i_). Values of ε_i_ close to 1 indicate linear increase in butterfly counts per hour effort. Values of ε_i_ close to zero indicate an asymptotic relationship where number of butterflies counted levels off with increasing sampling effort. (c) Map of monarch relative abundance trends ($\tau_{i}$) among grid cells. Cyan and pink highlighting denotes estimates whose 95% credible intervals were greater or less than zero, respectively. Maps of (d) glyphosate effect estimates ($\rho_{i}$), (e) cumulative precipitation effect estimates ($\nu_{i}$), and (f) mean temperature effect estimates ($\zeta_{i}$) among grid cells. For (d–e), cyan and pink highlighting denotes estimates whose 95% credible intervals were greater or less than zero, respectively.

Considering all available NABA data for monarchs across the entire breeding range in eastern and western North America, the median of posterior distributions for relative abundance trends ($\tau_{i}$) pooled across all grid cells suggested an overall annual increase in monarch relative abundance of 1.36% per year. However, there was an 84% chance of the global trend being >0 and a 16% chance of the global trend <0 (Figure S2). Cell‐specific relative abundance trends were generally the most negative in the US Northeast, parts of the Midwest, and in northwest California, and were generally the most positive in the US Southeast and Northwest (Figure 2a). Only 11 of the 334 grid cells exhibited relative abundance trends whose 95% credible intervals did not overlap zero, 10 of which were positive trends in Florida (Figure 2a). Relative abundance in 2018 ($\alpha_{i}$) was highest in the Midwest, and lowest in the Southeast (Figure 2b), generally consistent with what is considered to be the main breeding range of monarch butterflies during the seasonal times of most NABA counts (Jepsen et al., 2015). The increase in expected monarch counts per hour of sampling effort ($\epsilon_{i}$) was nearly linear ($\epsilon_{i}∼1$) throughout the Midwest and parts of the Northeast where monarchs are more abundant, while smaller values of $\epsilon_{i}$ in the West and much of the Southeast indicated near saturation of sampling space (Figure 2c), as expected in areas where monarchs are not as abundant.

The effect of glyphosate use on monarch relative abundance was generally negative, especially in the Midwest, where the negative effects in 27 grid cells exhibited 95% credible intervals that did not overlap zero (Figure 2d). Effects of cumulative precipitation varied spatially from positive to negative, but only one grid cell exhibited a significant local negative effect where the 95% credible interval did not overlap zero (Figure 2e). Effects of mean temperature also varied spatially, with negative effects in warmer locations and positive effects in colder locations. Temperature effects were most pronounced in the Midwest, where positive effects in 43 grid cells exhibited 95% credible intervals that did not overlap zero (Figure 2f).

## 4 DISCUSSION

Our analysis of the North American Butterfly Association's citizen‐science data from summer monitoring at 403 sites distributed across the United States and southern Canada suggests that the collective *breeding* population of monarchs in North America is not showing strong evidence of widespread declines. Rather, decreases in adult monarchs were apparent in parts of the Southwest, Northeast, and Corn Belt regions of the United States (Illinois, Indiana, Ohio, southern Wisconsin), while increases were evident throughout much of the US Northwest, Upper Midwest, and Southeast. The lack of strong relative‐abundance trends, particularly in the US Midwest, could be partly attributed to opposing effects of increased agricultural glyphosate use and increased ambient temperature due to climate change, where negative effects of glyphosate appeared to be offset by positive effects of temperature.

The lack of strong trends in the core breeding range is in contrast to studies that focus on winter colony size as measures of population abundance, where there are clearly multi‐decadal declines (Brower et al., 2012; Oberhauser et al., 2017; Semmens et al., 2016; Thogmartin, Wiederholt, et al., 2017; Zylstra et al., 2021), but is in general agreement with various breeding season and fall migration studies that have shown high variability in monarch abundance trends (Table S1). For example, Zylstra et al. (2021) examined trends in breeding monarch abundance using multiple citizen‐science datasets from the Midwest region. Their work showed modest declines of adult monarchs in that region, consistent with our findings. Meanwhile Ethier (2020) examined temporal trends in migrating monarch abundance for the southern Ontario region, and concluded there was no recent decline in the annual migratory cohort there, which is at the beginning of the migratory journey. Similarly, Culbertson et al. (2021) found no evidence of declines over 30 years in the number of migrating monarchs in the Atlantic coast region. Our analysis considering NABA counts from the entire breeding population suggests that monarchs may have some ability to rebound from winter declines during the breeding season, perhaps providing some counteracting upward movement of monarch numbers despite declines in the winter.

Even though the most recent evidence indicates the monarchs west of the Rocky Mountains should not be considered a separate population (Freedman et al., 2021; Talla et al., 2020), the assessment of monarch abundance in the west has traditionally been via counts of wintering monarchs along the California coast (Espeset et al., 2016; Pelton et al., 2019). However, as we found with the larger cohort of monarchs east of the Rockies, the trend of diminishing wintering colonies in California does not appear to mirror long‐term trends in breeding monarch abundance to the north or northeast, either in Oregon or Idaho (Figure 2a). In fact, the (admittedly) few locations with long‐term data in that region indicate an overall increasing trend (sampling the posterior distributions of $\tau_{i}$ for grid cells overlapping Oregon and Washington revealed an 86.7% probability that the monarch relative abundance trend was >0). However, we do note that the NABA data we had access to ended in 2018, before a dramatic drop in colony size in the winter of 2020/2021 (Crone & Schultz, 2021; James, 2021), so we do not know how this may (or may not) have affected breeding monarchs in the northwest. We also note that the NABA data showed a small region in central California where summer numbers are declining, which is consistent with other long‐term surveys from that same region (Espeset et al., 2016), and this consistency provides confidence in the NABA data. Further, reasons for declines in wintering monarchs in California have been the subject of ongoing debate, with some speculating that western monarchs may be transitioning to a less migratory lifestyle in California, which is being fueled by homeowner plantings of non‐native milkweed that thrives year‐round (Davis, 2022; James et al., 2022). Regardless of the reason, the discrepancy between wintering numbers and breeding abundance in the west, like that of the east, argues that overall population assessments should be based on multiple sources, and from across life stages.

Our analysis indicated that glyphosate use, while an important contributor to local monarch numbers, is significantly affecting only a portion of the summer breeding range (portions of upper Midwest, Figure 2d). The initial rapid increase in glyphosate use in midwestern corn and soybean, which likely devastated weedy milkweeds in those fields, has now leveled (Zylstra et al., 2021), such that harmful indirect effects of herbicides on monarchs may no longer be increasing in magnitude. This suggests that the loss of agricultural milkweed in the US Midwest will not inevitably lead to ongoing drops in summertime abundances (Agrawal & Inamine, 2018). In fact, a recent inventory of the western half of the United States revealed billions of previously uncounted native milkweeds that are available for monarchs (Spaeth et al., 2022), supporting the notion that there are sufficient hostplants to maintain a stable summer population throughout much of the breeding range.

Recent analyses indicate that changing climate is driving increases and decreases in overall butterfly numbers across North America (Crossley et al., 2021; Forister et al., 2021), and, there is evidence that temperature and precipitation in North America is indirectly and positively impacting abundances of overwintering monarchs, via positive effects on breeding monarch population size (Zylstra et al., 2021). Such counterbalancing effects of seasonal temperature and precipitation appear to be common in butterflies (Davies, 2019; Konvicka et al., 2021; Roland & Matter, 2016). In line with this, we found a pattern of increasing monarch relative abundance with increasing average temperature in the northern US, with the strongest effects evident in the midwestern US (Figure 2f), where glyphosate use appeared to have the strongest negative effect (Figure 2d). Positive and negative effects of precipitation were also evident, but this signal was statistically less robust (Figure 2e). The eastern US and Canada, the area corresponding to the major monarch summer breeding ground for the eastern subpopulation, have generally seen increases in precipitation and only modest increases in summer temperature (IPCC, 2018), conditions that have apparently been providing favorable conditions for many butterfly species (Crossley et al., 2021). However, Texas and the northern portions of Mexico, a vital corridor region, have seen recent pronounced increases in temperatures (Cuervo‐Robayo et al., 2020) which could be negatively affecting survivorship during the arduous southward migration.

Overall, our findings suggest monarch populations may have some ability to recover, on average, from declines at overwintering colonies. Of course, the total loss of overwintering monarchs would make it impossible for any summer rebound to be ignited, and there almost certainly is some inflection point well before total winter extinction where spring migrants would be too few to reliably spark a summer resurgence. This would leave only the year‐round resident monarch populations, with the loss of the epic migrations that inspire much human interest in monarchs as conservation icons. For those monarchs that do return northward in the spring, our results argue that following the winter period, monarchs experience high population growth, perhaps facilitated by reduced intraspecific competition among larvae. Indeed, monarch larvae are known to exhibit negative interactions with conspecifics, including egg cannibalization (Brower, 1961), aggression (Collie et al., 2020), and oviposition avoidance on optimal host plants (Jones & Agrawal, 2019), behaviors that are presumably reduced under the smaller population returning from recent years in winter migration. Considering the general lack of widespread breeding season declines found here, our evidence suggests, alongside the ongoing declines at winter colonies, that monarchs must be experiencing increasingly higher levels of mortality during their fall migration. Contrasting evidence of no change in the number of tagged monarchs returning to Mexico in the fall suggested otherwise (Taylor et al., 2020), but that finding remains contested due to difficulties in accounting for changing tagging effort through time (Fordyce et al., 2020). In support of our assessment, a recent study of the monarch parasite, *Ophryocystis elektroscirrha*, has shown that nation‐wide prevalence has increased in the last 15 years, and that this increase is leading to considerable migratory losses and corresponding reductions in winter colony sizes (Majewska et al., 2021). Therefore, conservation attention along the migration routes, and/or actions that reduce parasite transmission, may be more imperative for the monarch's long‐term survival compared to efforts directed at the breeding grounds.

Our data were collected by citizen‐scientists, a method that requires careful use (Burgess et al., 2017), but that nonetheless enables inquiry at spatiotemporal scales otherwise unachievable by individual research groups (e.g., Herremans et al., 2021). The number of party hours spent monitoring butterflies in the North American Butterfly Association dataset increased on average by 1.2% (±0.3%) per year between 1993 and 2017 (Figure S3). However, our analyses accounted for annual variation in sampling effort while allowing for a variety of relationships between increasing sampling effort and monarch counts, following methods developed to analyze conceptually similar Audubon Christmas Bird Counts (Meehan et al., 2019). Importantly, we did not find evidence of increasing or decreasing trends in sampling effort around sites dominated by cropland or forest, suggesting that changes in sampling effort have neither masked declines nor exaggerated abundance increases (Figure S4). Furthermore, we found that the local effects of sampling effort exhibited an increasing impact on expected monarch counts in the Upper Midwest and Northeast (Figure 2c), suggesting that NABA counts are likely underestimating numbers of monarchs in the northern portion of their breeding range. This contrasts with the notion that NABA counts are spatially tracking a dwindling monarch population through the landscape, which would have yielded an asymptotic relationship between sampling effort and numbers of expected monarchs, as observed in the western and southern US.

**FIGURE 3:**
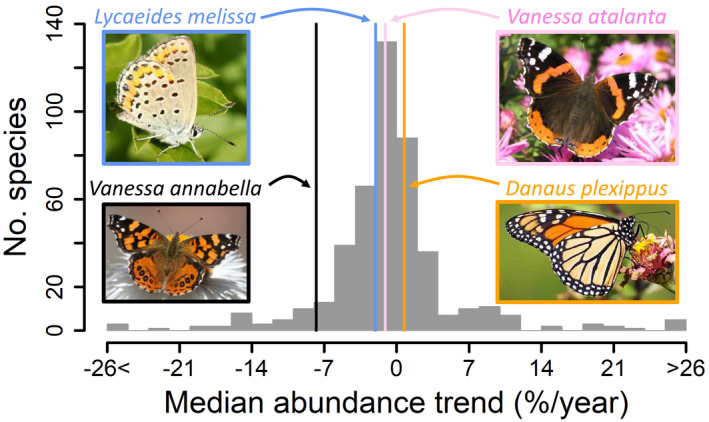
Monarch abundance trend compared with other common North American butterflies. Histogram depicts median abundance trends (%/year) of >450 species monitored by the North American Butterfly Association. Trend for *Danaus plexippus* (+0.7%/year) is highlighted and compared with three other well‐known species, *Lycaeides melissa* (−2.0%/year), *Vanessa annabella* (−7.8%/year), and *Vanessa atalanta* (−1.1%/year). Trends based on sites where butterflies were recorded at least five times over a span of 10 years. See Crossley et al. (2021) for details on trend estimation. All butterfly species trends are available in Table S6.

Beyond monarchs, the conservation of insects has received far less attention than most other taxa, despite the ubiquity of insects in terrestrial ecosystems. Undoubtedly, citizen‐science efforts targeting the charismatic monarch have exposed many non‐scientists in North America to the importance of insects and the value of their conservation. Given our results, we suggest that there could be considerable ecological gain from broadening citizen scientists' attention to also consider the many butterfly species that do appear to be experiencing major summer declines across North America. For example, the summer butterfly count data suggest that *Lycaeides melissa* is declining across much of its broad range (Figure 3), and even the well‐known west coast painted lady, *Vanessa annabella*, appears to be faring worse than the monarch (Figure 3). In fact, of the 456 butterfly species tracked by NABA, there are 320 species with trends less positive than monarch butterflies (Crossley et al., 2021). More broadly, our results are consistent with other recent analyses of large‐scale insect data that have also revealed complex and heterogeneous spatiotemporal patterns of insect decline. For example, a warming climate in Europe is shifting some moth ranges northward, with species unable to do so declining, but leading to a net range increase overall (Fox et al., 2021). Similarly, recent drops in UK moths seem modest relative to increases seen over prior decades (Macgregor et al., 2019), leading to no net change over time. In North America, close examination of long‐term insect counts revealed declines in some taxa, but increases in others (Crossley et al., 2020). The same is true with butterflies, where species declines in western North America may be at least partially offset by abundance increases elsewhere on the continent (Crossley et al., 2021), again, leading to no net change despite troubling declines in some locations and/or for some taxa. Our analyses show that for monarchs, for now, summer abundance increases appear sufficient to buffer winter declines. It will be increasingly important to understand complex interactions among species traits and mechanistic drivers, in order to understand and successfully predict how an ever‐more‐rapidly changing environment will impact the future persistence of monarchs and other insects.

## FUNDING INFORMATION

We acknowledge funding from USDA‐NIFA‐OREI 2015–51,300‐24,155, USDA‐NIFA‐SCRI 2015–51,181‐24,292, and USDA‐Hatch DEL00774.

## CONFLICT OF INTEREST

The authors declare no competing interests.

## Supporting information


**Appendix S1** Supporting Information

## Data Availability

Data (relative abundances and environmental covariates) and R code supporting the findings of this study are available on Dryad (DOI https://doi.org/10.5061/dryad.7pvmcvdwb). Raw data are available from the North American Butterfly Association (NABA) at https://www.naba.org/

## References

[gcb16282-bib-0001] Agrawal, A. A. , & Inamine, H. (2018). Mechanisms behind the monarch's decline. Science, 360(6395), 1294–1296. 10.1126/science.aat5066 29930122

[gcb16282-bib-0002] Bale, J. S. , Masters, G. J. , Hodkinson, I. D. , Awmack, C. , Bezemer, T. M. , Brown, V. K. , Butterfield, J. , Buse, A. , Coulson, J. C. , Farrar, J. , Good, J. E. G. , Harrington, R. , Hartley, S. , Jones, T. H. , Lindroth, R. L. , Press, M. C. , Symrnioudis, I. , Watt, A. D. , & Whittaker, J. B. (2002). Herbivory in global climate change research: Direct effects of rising temperature on insect herbivores. Global Change Biology, 8(1), 1–16. 10.1046/j.1365-2486.2002.00451.x

[gcb16282-bib-0003] Baxter‐Gilbert, J. H. , Riley, J. L. , Neufeld, C. J. H. , Litzgus, J. D. , & Lesbarrères, D. (2015). Road mortality potentially responsible for billions of pollinating insect deaths annually. Journal of Insect Conservation, 19(5), 1029–1035. 10.1007/s10841-015-9808-z

[gcb16282-bib-0004] Besag, J. , York, J. , & Mollie, A. (1991). Bayesian image restoration, with two applications in spatial statistics. Annals of the Institute of Statistical Mathemat‐ Ics, 43(1), 1–59.

[gcb16282-bib-0005] Bivand, R. , Keitt, T. , & Rowlingson, B. (2021). rgdal: Bindings for the “Geospatial” data abstraction library. https://cran.r‐project.org/package=rgdal

[gcb16282-bib-0006] Bivand, R. , & Rundel, C. (2021). rgeos: Interface to Geometry Engine ‐ Open Source ('GEOS’). https://cran.r‐project.org/package=rgeos

[gcb16282-bib-0007] Boyle, J. H. , Dalgleish, H. J. , & Puzey, J. R. (2019). Monarch butterfly and milkweed declines substantially predate the use of genetically modified crops. Proceedings of the National Academy of Sciences of the United States of America, 116(8), 3006–3011. 10.1073/pnas.1811437116 30723147 PMC6386695

[gcb16282-bib-0008] Brower, L. P. (1961). Experimental analyses of egg cannibalism in the Monarch and Queen butterflies, Danaus plexippus and D. gilippus berenice. Physiological Zoology, 34(4), 287–296.

[gcb16282-bib-0009] Brower, L. P. , Taylor, O. R. , Williams, E. H. , Slayback, D. A. , Zubieta, R. R. , & Ramírez, M. I. (2012). Decline of monarch butterflies overwintering in Mexico: Is the migratory phenomenon at risk? Insect Conservation and Diversity, 5(2), 95–100. 10.1111/j.1752-4598.2011.00142.x

[gcb16282-bib-0010] Brower, L. P. , Williams, E. H. , Dunford, K. S. , Dunford, J. C. , Knight, A. L. , Daniels, J. , Cohen, J. A. , Van Hook, T. , Saarinen, E. , Standridge, M. J. , Epstein, S. W. , Zalucki, M. P. , & Malcolm, S. B. (2018). A long‐term survey of spring monarch butterflies in north‐central Florida. Journal of Natural History, 52(31–32), 2025–2046. 10.1080/00222933.2018.1510057

[gcb16282-bib-0011] Burgess, H. K. , DeBey, L. B. , Froehlich, H. E. , Schmidt, N. , Theobald, E. J. , Ettinger, A. K. , HilleRisLambers, J. , Tewksbury, J. , & Parrish, J. K. (2017). The science of citizen science: Exploring barriers to use as a primary research tool. Biological Conservation, 208, 113–120. 10.1016/j.biocon.2016.05.014

[gcb16282-bib-0012] Collie, J. , Granela, O. , Brown, E. B. , & Keene, A. C. (2020). Aggression is induced by resource limitation in the monarch caterpillar. IScience, 23(12), 101791. 10.1016/j.isci.2020.101791 33376972 PMC7756136

[gcb16282-bib-0013] Crone, E. E. , & Schultz, C. B. (2021). Resilience or Catastrophe? A possible state change for monarch butterflies in western North America. Ecology Letters, 24(8), 1533–1538. 10.1111/ele.13816 34110069

[gcb16282-bib-0014] Crossley, M. S. , Meier, A. R. , Baldwin, E. M. , Berry, L. L. , Crenshaw, L. C. , Hartman, G. L. , Lagos‐Kutz, D. , Nichols, D. H. , Patel, K. , Varriano, S. , Snyder, W. E. , & Moran, M. D. (2020). No net insect abundance and diversity declines across US Long Term Ecological Research sites. Nature Ecology and Evolution, 4(10), 1368–1376. 10.1038/s41559-020-1269-4 32778751

[gcb16282-bib-0015] Crossley, M. S. , Smith, O. M. , Berry, L. L. , Phillips‐Cosio, R. , Glassberg, J. , Holman, K. M. , Holmquest, J. G. , Meier, A. R. , Varriano, S. A. , McClung, M. R. , Moran, M. D. , & Snyder, W. E. (2021). Recent climate change is creating hotspots of butterfly increase and decline across North America. Global Change Biology, 27(12), 2702–2714. 10.1111/gcb.15582 33749964

[gcb16282-bib-0016] Cuervo‐Robayo, A. P. , Ureta, C. , Gómez‐Albores, M. A. , Meneses‐Mosquera, A. K. , Téllez‐Valdés, O. , & Martínez‐Meyer, E. (2020). One hundred years of climate change in Mexico. PLoS One, 15(7 July), 1–19. 10.1371/journal.pone.0209808 PMC7365465 32673306

[gcb16282-bib-0017] Culbertson, K. A. , Garland, M. S. , Walton, R. K. , Zemaitis, L. , & Pocius, V. M. (2021). Long‐term monitoring indicates shifting fall migration timing in monarch butterflies (Danaus plexippus). Global Change Biology, 28, 727–738. 34693598 10.1111/gcb.15957

[gcb16282-bib-0018] Davies, W. J. (2019). Multiple temperature effects on phenology and body size in wild butterflies predict a complex response to climate change. Ecology, 100(4), 1–11. 10.1002/ecy.2612 30636278

[gcb16282-bib-0019] Davis, A. K. (2022). Monarchs reared in winter in California are not large enough to be migrants. Comment on James et al. First population study on winter breeding monarch butterflies, *Danaus plexippus* (Lepidoptera: Nymphalidae) in the urban South Bay of San Francisco, California. Insects 2021, 12, 946. Insects, 13, 63. 35055906 10.3390/insects13010063 PMC8778705

[gcb16282-bib-0020] Davis, A. K. , & Dyer, L. A. (2015). Long‐term trends in eastern North American monarch butterflies: A collection of studies focusing on spring, summer, and fall dynamics. Annals of the Entomological Society of America, 108(5), 661–663. 10.1093/aesa/sav070

[gcb16282-bib-0021] Didham, R. K. , Basset, Y. , Collins, C. M. , Leather, S. R. , Littlewood, N. A. , Menz, M. H. M. , Müller, J. , Packer, L. , Saunders, M. E. , Schönrogge, K. , Stewart, A. J. A. , Yanoviak, S. P. , & Hassall, C. (2020). Interpreting insect declines: seven challenges and a way forward. Insect Conservation and Diversity, 13(2), 103–114. 10.1111/icad.12408

[gcb16282-bib-0022] Espeset, A. E. , Harrison, J. G. , Shapiro, A. M. , Nice, C. C. , Thorne, J. H. , Waetjen, D. P. , Fordyce, J. A. , & Forister, M. L. (2016). Understanding a migratory species in a changing world: climatic effects and demographic declines in the western monarch revealed by four decades of intensive monitoring. Oecologia, 181(3), 819–830. 10.1007/s00442-016-3600-y 27000943

[gcb16282-bib-0023] Ethier, D. M. (2020). Population trends of monarch butterflies (Lepidoptera: Nymphalidae) migrating from the core of Canada's eastern breeding population. Annals of the Entomological Society of America, 113(6), 461–467. 10.1093/aesa/saaa021

[gcb16282-bib-0024] Flockhart, D. T. T. , Brower, L. P. , Ramirez, M. I. , Hobson, K. A. , Wassenaar, L. I. , Altizer, S. , & Norris, D. R. (2017). Regional climate on the breeding grounds predicts variation in the natal origin of monarch butterflies overwintering in Mexico over 38 years. Global Change Biology, 23(7), 2565–2576. 10.1111/gcb.13589 28045226

[gcb16282-bib-0025] Fordyce, J. A. , Nice, C. C. , & Forister, M. L. (2020). Commentary: Evaluating the migration mortality hypothesis using monarch tagging data. Frontiers in Ecology and Evolution, 8(November), 1–3. 10.3389/fevo.2020.00264

[gcb16282-bib-0026] Forister, M. L. , Halsch, C. A. , Nice, C. C. , Fordyce, J. A. , Dilts, T. E. , Oliver, J. C. , Prudic, K. L. , Shapiro, A. M. , Wilson, J. K. , & Glassberg, J. (2021). Fewer butterflies seen by community scientists across the warming and drying landscapes of the American West. Science, 371(6533), 1042–1045. 10.1126/science.abe5585 33674492

[gcb16282-bib-0027] Fox, R. , Dennis, E. B. , Harrower, C. A. , Blumgart, D. , Bell, J. R. , Cook, P. , Davis, A. M. , Evans‐Hill, L. J. , Haynes, F. , Hill, D. , & Isaac, N. J. (2021). The state of Britain's larger moths. Butterfly‐Conservation.Org. https://butterfly‐conservation.org/sites/default/files/2021‐03/StateofMothsReport2021.pdf

[gcb16282-bib-0028] Fox, R. (2013). The decline of moths in Great Britain: A review of possible causes. Insect Conservation and Diversity, 6(1), 5–19. 10.1111/j.1752-4598.2012.00186.x

[gcb16282-bib-0029] Freedman, M. G. , de Roode, J. C. , Forister, M. L. , Kronforst, M. R. , Pierce, A. A. , Schultz, C. B. , Taylor, O. R. , & Crone, E. E. (2021). Are eastern and western monarch butterflies distinct populations? A review of evidence for ecological, phenotypic, and genetic differentiation and implications for conservation. Conservation Science and Practice, 3(7). 10.1111/csp2.432

[gcb16282-bib-0030] Gustafsson, K. M. , Agrawal, A. A. , Lewenstein, B. V. , & Wolf, S. A. (2015). The monarch butterfly through time and space: The social construction of an icon. Bioscience, 65(6), 612–622. 10.1093/biosci/biv045

[gcb16282-bib-0031] Habel, J. C. , Samways, M. J. , & Schmitt, T. (2019). Mitigating the precipitous decline of terrestrial European insects: Requirements for a new strategy. Biodiversity and Conservation, 28(6), 1343–1360. 10.1007/s10531-019-01741-8

[gcb16282-bib-0032] Habel, J. C. , Ulrich, W. , Biburger, N. , Seibold, S. , & Schmitt, T. (2019). Agricultural intensification drives butterfly decline. Insect Conservation and Diversity, 12(4), 289–295. 10.1111/icad.12343

[gcb16282-bib-0033] Harris, I. , Jones, P. D. , Osborn, T. J. , & Lister, D. H. (2014). Updated high‐resolution grids of monthly climatic observations ‐ the CRU TS3.10 Dataset. International Journal of Climatology, 34(3), 623–642. 10.1002/joc.3711

[gcb16282-bib-0035] Herremans, M. , Gielen, K. , Van Kerckhoven, J. , Vanormelingen, P. , Veraghtert, W. , Swinnen, K. R. R. , & Maes, D. (2021). Abundant citizen science data reveal that the peacock butterfly *Aglais io* recently became bivoltine in Belgium. Insects, 12(8). 10.3390/insects12080683 PMC8396639 34442249

[gcb16282-bib-0036] Hijmans, R. J. (2022). raster: Geographic data analysis and modeling. https://cran.r‐project.org/package=raster

[gcb16282-bib-0037] Inamine, H. , Ellner, S. P. , Springer, J. P. , & Agrawal, A. A. (2016). Linking the continental migratory cycle of the monarch butterfly to understand its population decline. Oikos, 125(8), 1081–1091. 10.1111/oik.03196

[gcb16282-bib-0038] IPCC . (2018). IPCC Special Report on the impacts of global warming of 1.5°C. IPCC ‐ Sr15, 2(October), 17–20. www.environmentalgraphiti.org

[gcb16282-bib-0039] James, D. G. (2021). Western North American monarchs: Spiraling into oblivion or adapting to a changing environment? Animal Migration, 8(1), 19–26. 10.1515/ami-2021-0002

[gcb16282-bib-0040] James, D. G. , Schaefer, M. C. , Easton, K. K. , & Carl, A. (2022). Reply to Davis, A.K. Monarchs reared in winter in California are not large enough to be migrants. Comment on “James et al. First population study on winter breeding monarch butterflies, Danaus plexippus (Lepidoptera: Nymphalidae) in the urban South Bay ofSouth Bay of San Francisco, California. Insects 2021, 12, 946”. Insects, 13, 64. 35055907 10.3390/insects13010064 PMC8777960

[gcb16282-bib-0041] Jamieson, M. A. , Trowbridge, A. M. , Raffa, K. F. , Lindroth, R. L. , & Wisconsin, M. A. J. (2012). Consequences of climate warming and altered precipitation patterns for plant‐insect and multitrophic interactions. Plant Physiology, 160, 1719–1727. 10.1104/pp.112.206524 23043082 PMC3510105

[gcb16282-bib-0042] Jepsen, S. , Schweitzer, D. F. , Young, B. , & Sears, N. (2015). Conservation status and ecology of the monarch butterfly in the United States. *The Xerces Society for Intvertebrate Conservation* . https://www.xerces.org/wp‐content/uploads/2015/03/NatureServe‐Xerces_monarchs_USFS‐final.pdf

[gcb16282-bib-0043] Jones, P. L. , & Agrawal, A. A. (2019). Beyond preference and performance: host plant selection by monarch butterflies, Danaus plexippus. Oikos, 128, 1092–1102. 10.1111/oik.06001

[gcb16282-bib-0044] Kantola, T. , Tracy, J. L. , Baum, K. A. , Quinn, M. A. , & Coulson, R. N. (2019). Spatial risk assessment of eastern monarch butterfly road mortality during autumn migration within the southern corridor. Biological Conservation, 231(December 2018), 150–160. 10.1016/j.biocon.2019.01.008

[gcb16282-bib-0045] Koltz, A. M. , Schmidt, N. M. , & Høye, T. T. (2018). Differential arthropod responses to warming are altering the structure of arctic communities. Royal Society Open Science, 5(4). 10.1098/rsos.171503 PMC5936898 29765633

[gcb16282-bib-0046] Konvicka, M. , Kuras, T. , Liparova, J. , Slezak, V. , Horázná, D. , Kleèka, J. , & Kleckova, I. (2021). Low winter precipitation, but not warm autumns and springs, threatens mountain butterflies in middle‐high mountains. PeerJ, 9, 1–23. 10.7717/peerj.12021 PMC8404571 34532158

[gcb16282-bib-0047] Leather, S. R. (2018). “Ecological Armageddon” – more evidence for the drastic decline in insect numbers. Annals of Applied Biology, 172(1), 1–3. 10.1111/aab.12410

[gcb16282-bib-0048] Lemoine, N. P. (2015). Climate change may alter breeding ground distributions of eastern migratory monarchs (Danaus plexippus) via range expansion of Asclepias host plants. PLoS One, 10(2), e0118614. 10.1371/journal.pone.0118614 25705876 PMC4338007

[gcb16282-bib-0049] Lindgren, F. , & Rue, H. (2015). Bayesian Spatial Modelling with R. INLA, 63(19), 1–25.

[gcb16282-bib-0050] Link, W. A. , & Sauer, J. R. (1999). Controlling for varying effort in count survey: An analysis of Christmas Bird Count data. Journal of Agricultural, Biological, and Environmental Statistics, 4(2), 116–125.

[gcb16282-bib-0051] Macgregor, C. J. , Williams, J. H. , Bell, J. R. , & Thomas, C. D. (2019). Moth biomass increases and decreases over 50 years in Britain. Nature Ecology and Evolution, 3(12), 1645–1649. 10.1038/s41559-019-1028-6 31712694

[gcb16282-bib-0052] Majewska, A. A. , Davis, A. K. , Altizer, S. , & Roode, J. C. (2021). Parasite dynamics in North American monarchs predicted by host density and seasonal migratory culling. Journal of Animal Ecology, 2022, 1–14. 10.1111/1365-2656.13678 35174493

[gcb16282-bib-0053] McKenna, D. D. , McKenna, K. M. , Malcom, S. B. , & Berenbaum, M. R. (2001). Mortality of lepidoptera along roadways in Central Illinois. Journal of the Lepidopterists' Society, 55(2), 63–68.

[gcb16282-bib-0054] Meehan, T. D. , Michel, N. L. , & Rue, H. (2019). Spatial modeling of Audubon Christmas Bird Counts reveals fine‐scale patterns and drivers of relative abundance trends. Ecosphere, 10(4), e02707. 10.1002/ecs2.2707

[gcb16282-bib-0055] Oberhauser, K. , Wiederholt, R. , Diffendorfer, J. E. , Semmens, D. , Ries, L. , Thogmartin, W. E. , Lopez‐Hoffman, L. , & Semmens, B. (2017). A trans‐national monarch butterfly population model and implications for regional conservation priorities. Ecological Entomology, 42(1), 51–60. 10.1111/een.12351

[gcb16282-bib-0056] Olaya‐Arenas, P. , & Kaplan, I. (2019). Quantifying pesticide exposure risk for monarch caterpillars on milkweeds bordering agricultural land. Frontiers in Ecology and Evolution, 7(JUN), 1–16. 10.3389/fevo.2019.00223

[gcb16282-bib-0057] Owens, A. C. S. , Cochard, P. , Durrant, J. , Farnworth, B. , Perkin, E. K. , & Seymoure, B. (2020). Light pollution is a driver of insect declines. Biological Conservation, 241(August), 108259. 10.1016/j.biocon.2019.108259

[gcb16282-bib-0058] Pelton, E. M. , Schultz, C. B. , Jepsen, S. J. , Black, S. H. , & Crone, E. E. (2019). Western monarch population plummets: Status, probable causes, and recommended conservation actions. Frontiers in Ecology and Evolution, 7, 1–7. 10.3389/fevo.2019.00258

[gcb16282-bib-0059] Pleasants, J. M. , & Oberhauser, K. S. (2013). Milkweed loss in agricultural fields because of herbicide use: Effect on the monarch butterfly population. Insect Conservation and Diversity, 6(2), 135–144. 10.1111/j.1752-4598.2012.00196.x

[gcb16282-bib-0060] Potter, N. A. (2019). rnassqs: An R package to access agricultural data via the USDA National Agricultural Statistics Service. Journal of Open Source Software, 4, 1–3. 10.21105/joss.01880

[gcb16282-bib-0061] R Core Team . (2021). R: A language and environment for statistical computing. https://www.r‐project.org/

[gcb16282-bib-0062] Ries, L. , Taron, D. J. , & Rendón‐Salinas, E. (2015). The disconnect between summer and winter monarch trends for the eastern migratory population: Possible links to differing drivers. Annals of the Entomological Society of America, 108(5), 691–699. 10.1093/aesa/sav055

[gcb16282-bib-0063] Roland, J. , & Matter, S. F. (2016). Pivotal effect of early‐winter temperatures and snowfall on population growth of alpine *Parnassius smintheus* butterflies. Ecological Monographs, 86(4), 412–428. 10.1002/ecm.1225

[gcb16282-bib-0034] Rue, H. , Riebler, A. , Sørbye, S. H. , Illian, J. B. , Simpson, D. P. , & Lindgren, F. K. (2017). Bayesian computing with INLA: A review. Annual Review of Statistics and its Application, 4, 395–421. 10.1146/annurev-statistics-060116-054045

[gcb16282-bib-0064] Sánchez‐Bayo, F. , & Wyckhuys, K. A. G. (2019). Worldwide decline of the entomofauna: A review of its drivers. Biological Conservation, 232, 8–27. 10.1016/j.biocon.2019.01.020

[gcb16282-bib-0065] Saunders, S. P. , Ries, L. , Neupane, N. , Ramírez, M. I. , & García‐serrano, E. (2019). Multiscale seasonal factors drive the size of winter monarch colonies. Proceedings of the National Academy of Sciences of the United States of America, 116(17), 8609–8614. 10.1073/pnas.1805114116 30886097 PMC6486777

[gcb16282-bib-0066] Saunders, S. P. , Ries, L. , Oberhauser, K. S. , Thogmartin, W. E. , & Zipkin, E. F. (2018). Local and cross‐seasonal associations of climate and land use with abundance of monarch butterflies. 278–290. 10.1111/ecog.02719

[gcb16282-bib-0067] Semmens, B. X. , Semmens, D. J. , Thogmartin, W. E. , Wiederholt, R. , López‐Hoffman, L. , Diffendorfer, J. E. , Pleasants, J. M. , Oberhauser, K. S. , & Taylor, O. R. (2016). Quasi‐extinction risk and population targets for the Eastern, migratory population of monarch butterflies (Danaus plexippus). Scientific Reports, 6(February 2016), 1–7. 10.1038/srep23265 26997124 PMC4800428

[gcb16282-bib-0068] Simpson, D. , Rue, H. , Riebler, A. , Martins, T. G. , & Sørbye, S. H. (2017). Penalising model component complexity: A principled, practical approach to constructing priors, 32, 1–28. 10.1214/16-STS576

[gcb16282-bib-0069] Spaeth, K. E. , Barbour, P. J. , Moranz, R. , Dinsmore, S. J. , & Williams, C. J. (2022). *Asclepias* dynamics on US rangelands: implications for conservation of monarch butterflies and other insects. Ecosphere, 13(1). 10.1002/ecs2.3816

[gcb16282-bib-0070] Stenoien, C. , Nail, K. R. , Zalucki, J. M. , Parry, H. , Oberhauser, K. S. , & Zalucki, M. P. (2018). Monarchs in decline: A collateral landscape‐level effect of modern agriculture. Insect Sci., 25, 528–541. 10.1111/1744-7917.12404 27650673

[gcb16282-bib-0071] Talla, V. , Pierce, A. A. , Adams, K. L. , de Man, T. J. B. , Nallu, S. , Villablanca, F. X. , Kronforst, M. R. , & de Roode, J. C. (2020). Genomic evidence for gene flow between monarchs with divergent migratory phenotypes and flight performance. Molecular Ecology, 29(14), 2567–2582. 10.1111/mec.15508 32542770 PMC8118118

[gcb16282-bib-0072] Taylor, O. R. , Pleasants, J. M. , Grundel, R. , Pecoraro, S. D. , Lovett, J. P. , & Ryan, A. (2020). Evaluating the migration mortality hypothesis using monarch tagging data. Frontiers in Ecology and Evolution, 8(August), 1–13. 10.3389/fevo.2020.00264

[gcb16282-bib-0073] Thogmartin, W. E. , López‐hoffman, L. , Rohweder, J. , Diffendorfer, J. , Drum, R. , Semmens, D. , Black, S. , Caldwell, I. , Cotter, D. , Drobney, P. , Jackson, L. L. , Gale, M. , Helmers, D. , Hilburger, S. , Howard, E. , & Oberhauser, K. (2017). Restoring monarch butterfly habitat in the Midwestern US: ‘all hands on deck’. Environmental Research Letters, 12, 074005.

[gcb16282-bib-0074] Thogmartin, W. E. , Wiederholt, R. , Oberhauser, K. , Drum, R. G. , Diffendorfer, J. E. , Altizer, S. , Taylor, O. R. , Pleasants, J. , Semmens, D. , Semmens, B. , Erickson, R. , Libby, K. , & Lopez‐Hoffman, L. (2017). Monarch butterfly population decline in North America: Identifying the threatening processes. Royal Society Open Science, 4(9). 10.1098/rsos.170760 PMC5627118 28989778

[gcb16282-bib-0075] USDA‐NASS . (2022). Quick Stats. https://quickstats.nass.usda.gov/

[gcb16282-bib-0076] USFWS . (2020). United States fish and wildlife service website ‐ Assessing the status of the monarch butterfly.

[gcb16282-bib-0077] USGS . (2022). Estimated annual agricultural pesticide use. Pesticide national synthesis project. https://water.usgs.gov/nawqa/pnsp/usage/maps/county‐level/

[gcb16282-bib-0078] Wagner, D. L. (2020). Insect declines in the Anthropocene. Annual Review of Entomology, 65, 457–480. 10.1146/annurev-ento-011019-025151 31610138

[gcb16282-bib-0079] Wilcove, D. S. , & Wikelski, M. (2008). Going, going, gone: Is animal migration disappearing? PLoS Biology, 6(7), e188. 10.1371/journal.pbio.0060188 18666834 PMC2486312

[gcb16282-bib-0080] Zylstra, E. R. , Ries, L. , Neupane, N. , Saunders, S. P. , Ramírez, M. I. , Rendón‐Salinas, E. , Oberhauser, K. S. , Farr, M. T. , & Zipkin, E. F. (2021). Changes in climate drive recent monarch butterfly dynamics. Nature Ecology and Evolution, 5(10), 1441–1452. 10.1038/s41559-021-01504-1 34282317

