# Supplementary material for: Opposing global change drivers counterbalance trends in breeding North American monarch butterflies
Source: Glob Chang Biol. 2022 Jun 10;28(15):4726–35. doi: 10.1111/gcb.16282 (PMC9542617; doi:10.1111/gcb.16282)
Supplement: Supplementary file 1 — Appendix S1 Supporting Information [file GCB-28-4726-s001.docx]

**Supplemental Information**

**Opposing global and local drivers counterbalance declines in breeding North American monarch butterflies**

Michael S. Crossley, Timothy D. Meehan, Matthew D. Moran, Jeffrey Glassberg, William E. Snyder, and Andrew K. Davis

**Supplemental Tables**

**Table S1.** Summary of reported trends in monarch abundance during spring migration, summer breeding, and fall migration.

| Season | Source | Data | Approach | Notes |
| --- | --- | --- | --- | --- |
| Spring | (Inamine et al., 2016) | Number of monarchs – NABA reports from Southern and Midwestern US in early spring; 2005-2014 | Model selection based on linear combinations of 'recipient', 'donor', 'year', and year x donor interaction | No evidence of temporal trends in numbers in "Midwest", "Northeast", Peninsula Point, or Cape May |
|  | (Brower et al., 2018) | Number of monarchs counted in the same pasture in Florida each year; 1994-2017 | Linear regression of yearly averages (monarchs/hr) on yea. | -4.9% adults/hr. No correlation between abundance in northern FL and previous year's numbers in Mexico |
| Summer | (Wepprich et al., 2019) | Number of monarchs - Ohio Butterfly Monitoring Program; 1996-2016 | GLMMs, Poisson distribution | -6.8%/yr; p-value = 0.002 |
|  | (Espeset et al., 2016) | Monarch presence / absence; 1972-2014 | binomial hierarchical Bayesian models | Odds ratio of observing a monarch decreased by a factor of 0.55/yr |
| Fall | (Crewe & McCracken, 2015; Ethier, 2020) | Average number of monarchs; 1995-2014 | log-linear regression using INLA (Bayesian) | Crewe & McCracken 2015: -5.11%/yr (CI: -0.97, -0.2); Ethier 2020: -5.25%/yr (CI: -8.60, -1.39) |
|  | (Michielini et al., 2021) | Number of monarchs/report - Massachusetts Butterfly Club data; 1992-2019 | GLMMs, Poisson distribution for counts, binomial for occurrence (list length used to account for variation in sampling effort) | Negligible trends for monarch occurrence (slopes not reported) |
|  | (Badgett & Davis, 2015) | Average number of monarchs flying over Peninsula Point, Michigan; 1996-2014 | GLM, monarch count log10-transformed | +9.6%/yr, p-value = 0.06 |
|  | (Ethier, 2020) | Number of roosts at Point Pelee, Ontario, Canada; 1984-2018 | log-linear regression (negative binomial) using INLA (Bayesian) | -0.69%/yr (CI: -5.35, -4.50) |
|  | (Culbertson et al., 2021) | Number of monarchs/hr at Cape May, New Jersey; 1992-2020 | linear regression of log-transformed yearly averages (monarchs/day) on year | -0.1%/yr, p-value = 0.976 |

**Supplemental Figures**


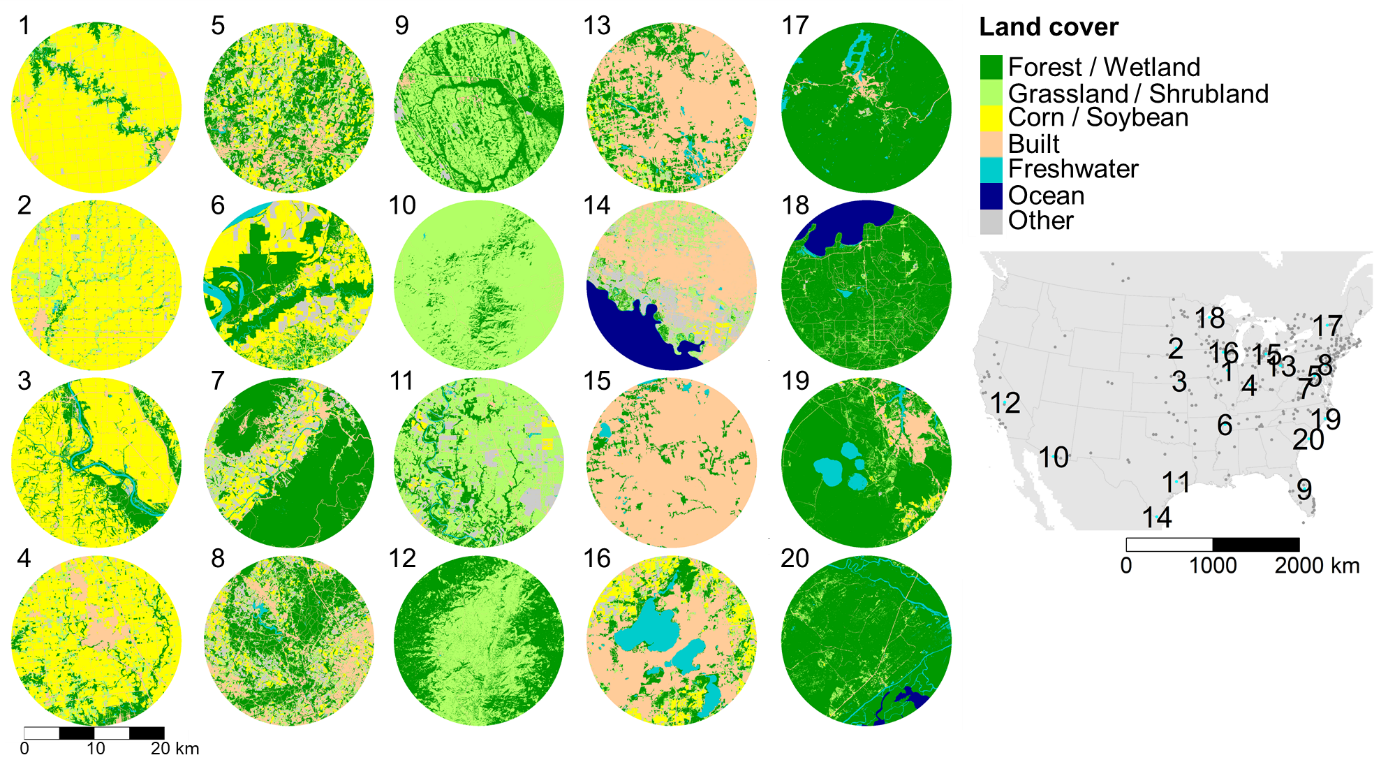


**Figure S1.** Landcover at selected NABA count sites, chosen to visualize the wide variation in landscapes covered by the program. Each circle is ~452 km^2^.


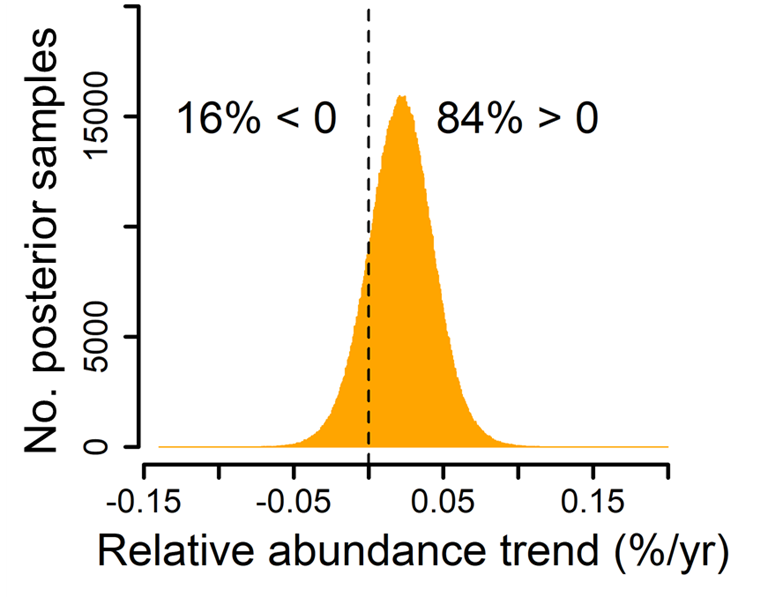


**Figure S2.** Histogram of posterior samples of $\tau$_i_ (monarch relative abundance trends), depicting the probability that the overall trend was < or > zero.

**
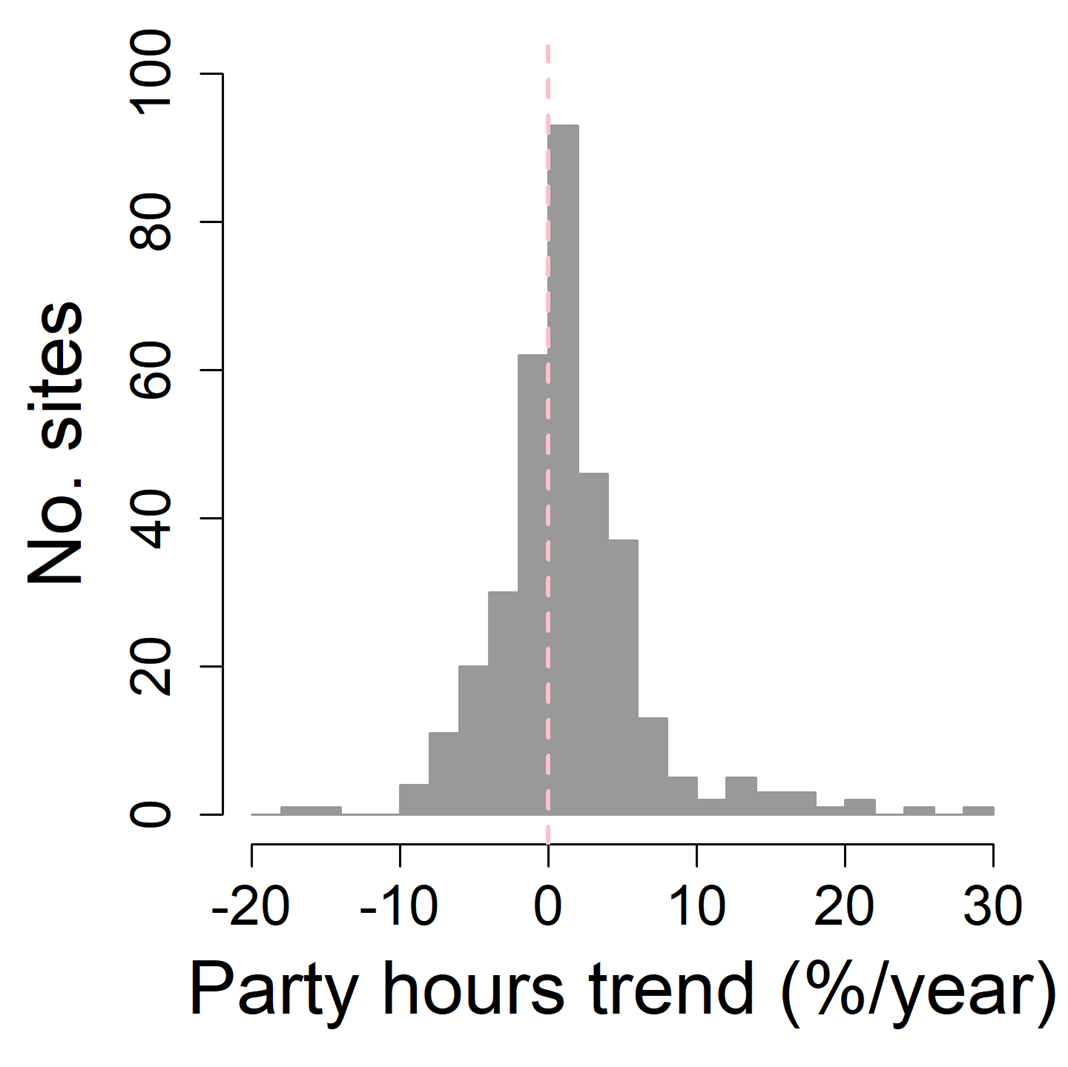
**

**Figure S3.** Trends in sampling effort among North American Butterfly Association (NABA) count circles. Histogram depicts trends (%/year) in party hours associated with butterfly counts at each NABA count circle.

**
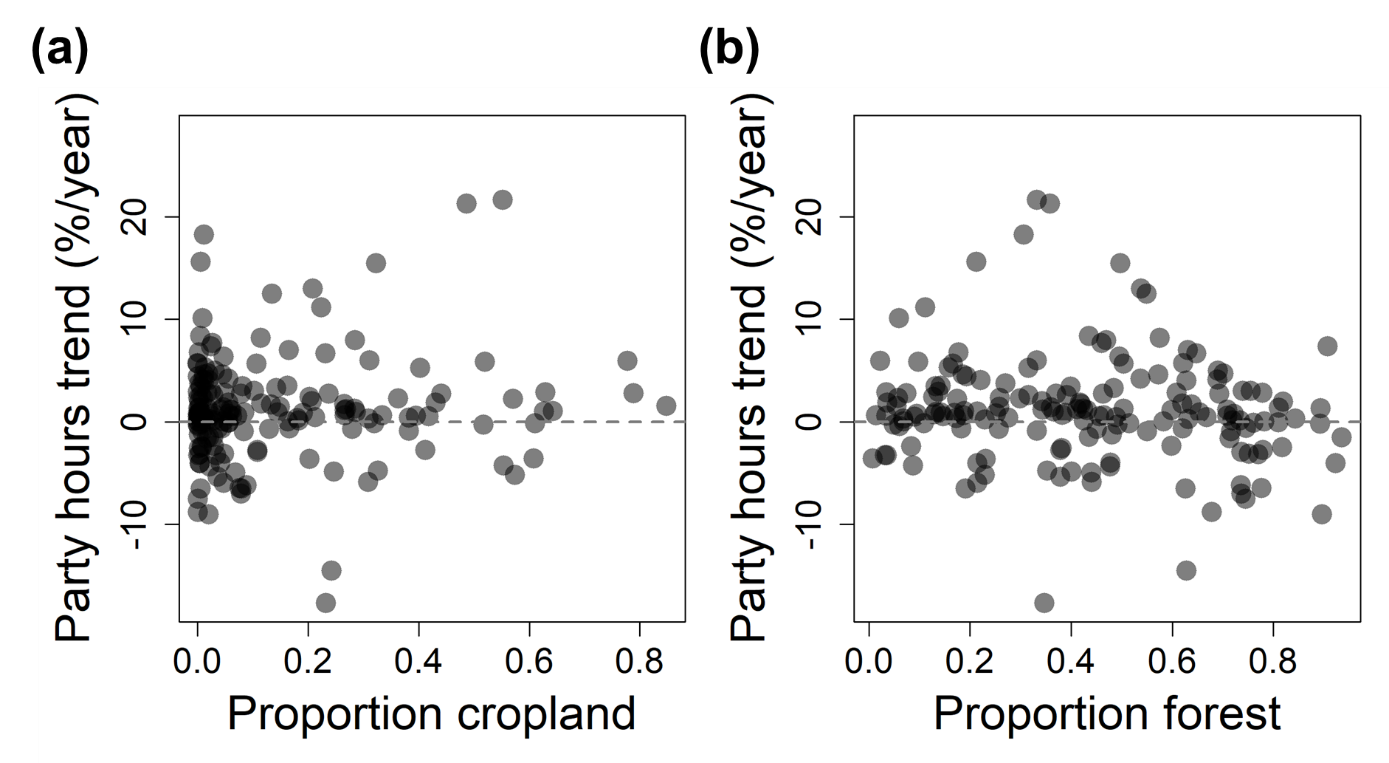
**

**Figure S4.** Lack of clustering of trends in sampling effort with land cover. **(a)** Scatterplot depicts the relationship between trend (%/year) in party hours and proportion cropland within ~24 km diameter North American Butterfly Association (NABA) count circles. **(b)** Scatterplot depicts the relationship between trend in party hours and proportion forest and wetland within NABA count circles.

**Literature cited**

Badgett, G., & Davis, A. K. (2015). Population trends of monarchs at a northern monitoring site: Analyses of 19 years of fall migration counts at peninsula point, MI. *Annals of the Entomological Society of America*, *108*(5), 700–706. https://doi.org/10.1093/aesa/sav060

Brower, L. P., Williams, E. H., Dunford, K. S., Dunford, J. C., Knight, A. L., Daniels, J., Cohen, J. A., Van Hook, T., Saarinen, E., Standridge, M. J., Epstein, S. W., Zalucki, M. P., & Malcolm, S. B. (2018). A long-term survey of spring monarch butterflies in north-central Florida. *Journal of Natural History*, *52*(31–32), 2025–2046. https://doi.org/10.1080/00222933.2018.1510057

Crewe, T. L., & McCracken, J. D. (2015). Long-term trends in the number of monarch butterflies (Lepidoptera: Nymphalidae) counted on fall migration at long point, Ontario, Canada (1995-2014). *Annals of the Entomological Society of America*, *108*(5), 707–717. https://doi.org/10.1093/aesa/sav041

Culbertson, K. A., Garland, M. S., Walton, R. K., Zemaitis, L., & Pocius, V. M. (2021). Long- term monitoring indicates shifting fall migration timing in monarch butterflies (*Danaus plexippus*). *Global Change Biology*, *28*, 727–738.

Espeset, A. E., Harrison, J. G., Shapiro, A. M., Nice, C. C., Thorne, J. H., Waetjen, D. P., Fordyce, J. A., & Forister, M. L. (2016). Understanding a migratory species in a changing world: climatic effects and demographic declines in the western monarch revealed by four decades of intensive monitoring. *Oecologia*, *181*(3), 819–830. https://doi.org/10.1007/s00442-016-3600-y

Ethier, D. M. (2020). Population trends of monarch butterflies (Lepidoptera: Nymphalidae) migrating from the core of Canada’s eastern breeding population. *Annals of the Entomological Society of America*, *113*(6), 461–467. https://doi.org/10.1093/aesa/saaa021

Inamine, H., Ellner, S. P., Springer, J. P., & Agrawal, A. A. (2016). Linking the continental migratory cycle of the monarch butterfly to understand its population decline. *Oikos*, *125*(8), 1081–1091. https://doi.org/10.1111/oik.03196

Michielini, J. P., Dopman, E. B., & Crone, E. E. (2021). Changes in flight period predict trends in abundance of Massachusetts butterflies. *Ecology Letters*, *24*, 249–257. https://doi.org/10.1111/ele.13637

Wepprich, T., Adrion, J. R., Ries, L., Wiedmann, J., Haddad, N. M., & Kellogg, W. K. (2019). Butterfly abundance declines over 20 years of systematic monitoring in Ohio, USA. *PLoS ONE*, *14*(7), e0216270. https://doi.org/10.1371/journal.pone.0216270
